# Supplementary material for: Automatic mapping of high-risk urban areas for Aedes aegypti infestation based on building facade image analysis
Source: PLoS Negl Trop Dis. 2024 Jun 3;18(6):e0011811. doi: 10.1371/journal.pntd.0011811 (PMC11192312; doi:10.1371/journal.pntd.0011811)
Supplement: Attachment — Submitted filename: Response to the Reviewers - Final.docx [file pntd.0011811.s001.docx]

**April 23, 2024**

**Manuscript Number: PNTD-D-23-01482**

**Automatic mapping of high-risk urban areas for *Aedes aegypti* infestation based on building facade image analysis**

**Dear Editor**,

In response to your letter regarding the manuscript mentioned above, we are submitting a detailed list of our responses to the review comments and a description of our changes. We are submitting two versions of our revised manuscript, one with tracked changes denoting where the text has been changed and the other a clean version. We have incorporated the suggestions provided by both referees into the revisions mentioned. Overall, we found their comments to be highly constructive and valuable. We extend our gratitude to you for giving us the opportunity to present a revised version of our manuscript. We also acknowledge the efforts made by all the reviewers to enhance the quality of our manuscript.

**Reviewer #1:**

**1. Vector control is the only option for reducing the incidence of dengue (from *Aedes aegypti*) for now, the development of meaningful surveillance methods is crucial. The PCINet model's low performance (less than 0.5 with 5-fold CV) raises concerns. Low accuracy and errors should not be overlooked, as even minor misclassifications can significantly alter risk assessments. The consideration of data augmentation, class weights, and sample weights in PCINet training is recommended. Data augmentation techniques such as rotation, flipping, scaling, and brightness adjustment can specifically address varying image qualities, including distortions such as blur and extreme lighting conditions of the collected samples described (section 3.2.3).**

**Answer:** We thank the reviewer for the insightful comment. Although accuracy alone suggests concerning results, we extensively edited section 4.2 to better portray the intended usage of our model. Since PCI, the output from PCINet, is a 5-point scale, with neighboring classes referring to very similar facade conditions, errors committed between nearby classes will hardly impact the identification of higher-risk areas in a large-scale scenario. We added a soft accuracy score along Section 4.2 and in Table 3, accounting for scenarios in which the absolute error, from a regression perspective, is equal to 1, considering it should not be treated equally as prediction errors between further classes. We also added the results for both recall and precision in Table 3, which allows for a better understanding of the results discussed in the paper.

This important observation made by the reviewer also inspired us to extensively edit the provided information for the second experiment with data from Google Street View (Sections 3.2.5 and created a novel Section 4.2.1) to include disagreement measures between annotators. This evidence supports our claim that absolute errors within a margin of 1 are acceptable if we consider human behavior a golden standard. This experiment allowed us to gather more annotations from the experts, which changed the amount of images we employed on the final test, as detailed in Section 3.2.5. We also took the opportunity to improve the maps from Figure 6, creating a new version that makes it easier to see the results. This was done by changing the color map of the data points and illustrating the results in the original 5-point scale schema of PCI instead of the 3-point scale proposed in the first version of the paper, which allows for the viewing of the differences between the 2 highest and 2 lowest PCI values, as well as being more adequate to the new evaluation in terms of a problem with a soft classification or regression.

Regarding the training procedure, we thank once more the reviewer for the wise suggestions. The focal loss, leveraged as optimization criteria in our work, allows us to provide class weights as indicated by the reviewer. Although we provided them in the first version of our work in the second paragraph of Section 4.2, we followed the suggestion also to include data augmentation techniques, which can indeed help to alleviate the problems presented in the original data. This is now introduced in the revised version thus the corresponding description was added in Section 4.2. Consequently, all metrics in section 4 are updated and images depicted in Figure 4 were resampled. Inspired by the observations provided by the reviewer, we also included one more step in the database filtering procedure described in Section 3.2.3, which allowed us to remove undesirable images, such as those in which the building's facade could not be seen, which can not be corrected or mitigated by data augmentation techniques.

**2. For a more comprehensive assessment of PCINet's practical utility and relevance, incorporating entomological data or disease case incidences into the evaluation is advised. The use of collected adult mosquito data with traps between two fieldworks (mentioned in section 3.2.2) is recommended.**

**Answer:** In the vector control program routine, we are constantly faced with the problem of the high proportion of pending properties (especially closed), which, in Campinas, is usually around 40 to 50%. This problem was accentuated by the fact that we conducted the surveys during the Covid-19 pandemic. These situations were considered when planning the work development to measure the PCI components to reach the number of properties necessary to be investigated.

For logistical reasons and the availability of human and material resources, it was impossible to install the traps while collecting information about the PCI. Thus, PCI measurement was conducted in two stages (between September to November 2021 and between March to May 2022), and the first trap installation cycle took place between December 2021 and February 2022, that is, between the two stages of PCI measurement. As a result, in many situations, the trap installation property did not coincide with the PCI measured nor with its closest neighbors, considering that a trap installed in a specific property would capture mosquitoes from this property and the nearest neighboring properties.

Since, in many situations, this concomitance did not occur, we chose to continue the study in a specific area of Campinas, installing traps and conducting the PCI measurement simultaneously to guarantee the non-occurrence of the same problem as before. Therefore, we are conducting a new research phase to compare these values, which still needs to be completed and will result in a new publication. Furthermore, there is a proposal for new studies to expand the capacity of PCINet to generate aggregated values, such as for blocks or larger aggregation areas. In this scenario, such comparison should be allowed, including the proposal to install traps to identify differences in infestation levels between areas.

**3. An extra parenthesis in "Facade condition)." in Table 1.**

**Answer:** We thank the reviewer for pointing out this mistake. This is now fixed in the new version of the paper.

**4. Need references to KartaView and Mapillary.**

**Answer:** We included the URL for both tools in footnotes. We thank the reviewer for this reminder.

**Reviewer #2:**

**1. The authors can try to explain why there is a strong relationship between the facade conditions and the building and backyard conditions.**

**Answer:** We thank the reviewer for providing such observations to improve our work. We included a discussion in the first paragraph of Section 5.1 – Discussion / Assumptions, explaining the strong relationship between the facade conditions and the building and backyard conditions.

**2. The authors mentioned they spend some efforts to collect mosquitos with adult traps. I wonder if they can analyze the relationsip between the mosquito(Aedes aegypti) numbers and the PCIs labled by human, so that the quality of the labled samples can be verified.**

**Answer:** In the vector control program routine, we are constantly faced with the problem of the high proportion of pending properties (especially closed), which, in Campinas, is usually around 40 to 50%. This problem was accentuated by the fact that we conducted the surveys during the Covid-19 pandemic. These situations were considered when planning the work development to measure the PCI components to reach the number of properties necessary to be investigated.

For logistical reasons and the availability of human and material resources, it was impossible to install the traps while collecting information about the PCI. Thus, PCI measurement was conducted in two stages (between September to November 2021 and between March to May 2022), and the first trap installation cycle took place between December 2021 and February 2022, that is, between the two stages of PCI measurement. As a result, in many situations, the trap installation property did not coincide with the PCI measured nor with its closest neighbors, considering that a trap installed in a specific property would capture mosquitoes from this property and the nearest neighboring properties.

Since, in many situations, this concomitance did not occur, we chose to continue the study in a specific area of Campinas, installing traps and conducting the PCI measurement simultaneously to guarantee the non-occurrence of the same problem as before. Therefore, we are conducting a new research phase to compare these values, which still needs to be completed and will result in a new publication. Furthermore, there is a proposal for new studies to expand the capacity of PCINet to generate aggregated values, such as for blocks or larger aggregation areas. In this scenario, such comparison should be allowed, including the proposal to install traps to identify differences in infestation levels between areas.

**3. I would like to suggest the authors make their sampled data and the source code of the presented PCINet model fully available.**

**Answer:** We thank the reviewer for the provided suggestions. The source code and trained models are now available on GitHub. Anyone interested can access it through the following link:

<https://github.com/patreo-lab/PCINet>

As for the sampled data, we can not make it openly available on the Internet due to it containing sensitive information regarding the condition of buildings from the inhabitants of Campinas. However, to allow for the reproducibility of our experiments and training of new models, we will evaluate the possibility of sending the data to those interested under the condition of filling out a form agreeing not to distribute the data and using it for scientific purposes only.
